# Supplementary material for: Soil moisture–atmosphere coupling accelerates global warming
Source: Nat Commun. 2023 Aug 15;14:4908. doi: 10.1038/s41467-023-40641-y (PMC10427638; doi:10.1038/s41467-023-40641-y)
Supplement: Supplementary file 1 — Supplementary Information [file 41467_2023_40641_MOESM1_ESM.pdf]

Soil moisture–atmosphere coupling accelerates global warming

Liang Qiao[1,3]†, Zhiyan Zuo[1,5,6]*†, Renhe Zhang[1]*, Shilong Piao[2], Dong Xiao[4], Kaiwen Zhang[1,3]

[1]Department of Atmospheric and Oceanic Sciences/Institute of Atmospheric Sciences, Fudan University, Shanghai, China.

[2]Sino-French Institute for Earth System Science, College of Urban and Environmental Sciences, Peking University, Beijing, China.

[3]State Key Laboratory of Severe Weather, Chinese Academy of Meteorological Sciences, Beijing, China.

[4]Key laboratory of Cites' Mitigation and Adaptation to Climate Change in Shanghai, China Meteorological Administration, Shanghai, China.

[5]National Observations and Research Station for Wetland Ecosystems of the Yangtze Estuary, Shanghai, China.

[6]Shanghai Frontiers Science Center of Atmosphere-Ocean Interaction, Shanghai, China.

*Corresponding author. Email: zuozhy@fudan.edu.cn; rhzhang@fudan.edu.cn

Contents of this Supplementary Information:

Supplementary Table 1

Supplementary Figures 1 to 11

Supplementary Table 1| Contributions to the warming trend (°C per decade).

The warming trend of total, soil moisture-atmosphere (SA), and greenhouse gases (GHG) effect on ground surface air temperature under high-emission scenario (SSP5-8.5) over globe (excluding Antarctica), northern middle latitudes (Northern: 30–60°N, 180°W–180°E), southern subtropical latitudes (Southern: 20–40°S, 180°W–180°E), Europe (EUR: 40–60°N, 20–50°E), and North America (NA: 28–55°N, 88–110°W) in future projections (2015-2099). The warming uncertainty is obtained by calculating the standard deviation of multiple models.

	Globe	Northern	Southern	EUR	NA
GHG	0.68±0.13	0.77±0.17	0.57±0.11	0.74±0.21	0.69±0.17
SA	0.05±0.03	0.09±0.05	0.03±0.02	0.17±0.08	0.16±0.09
Total	0.73±0.13	0.86±0.14	0.60±0.12	0.91±0.16	0.86±0.15

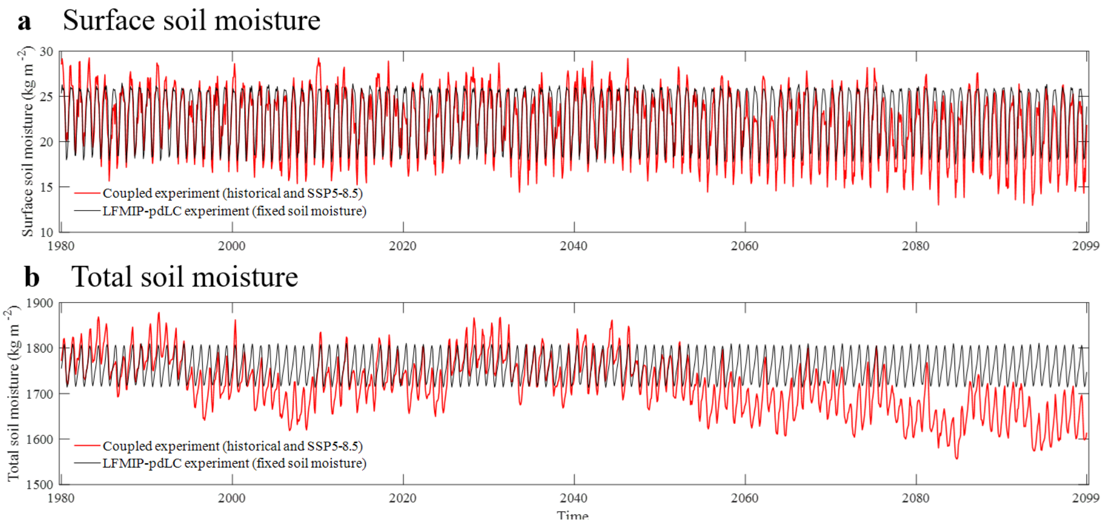

Supplementary Fig. 1| The multi-model mean of monthly soil moisture (kg m^{-2}) periods (1980–2099) for a grid in North America under high-emission scenario. a is surface soil moisture, and **b** is total soil moisture. Red lines represent fully coupled simulations (historical experiment in 1980-2014, and SSP5-8.5 experiment in 2015-2099); black lines represent fixed soil moisture simulations (LFMIP-pdLC in 1980-2099).

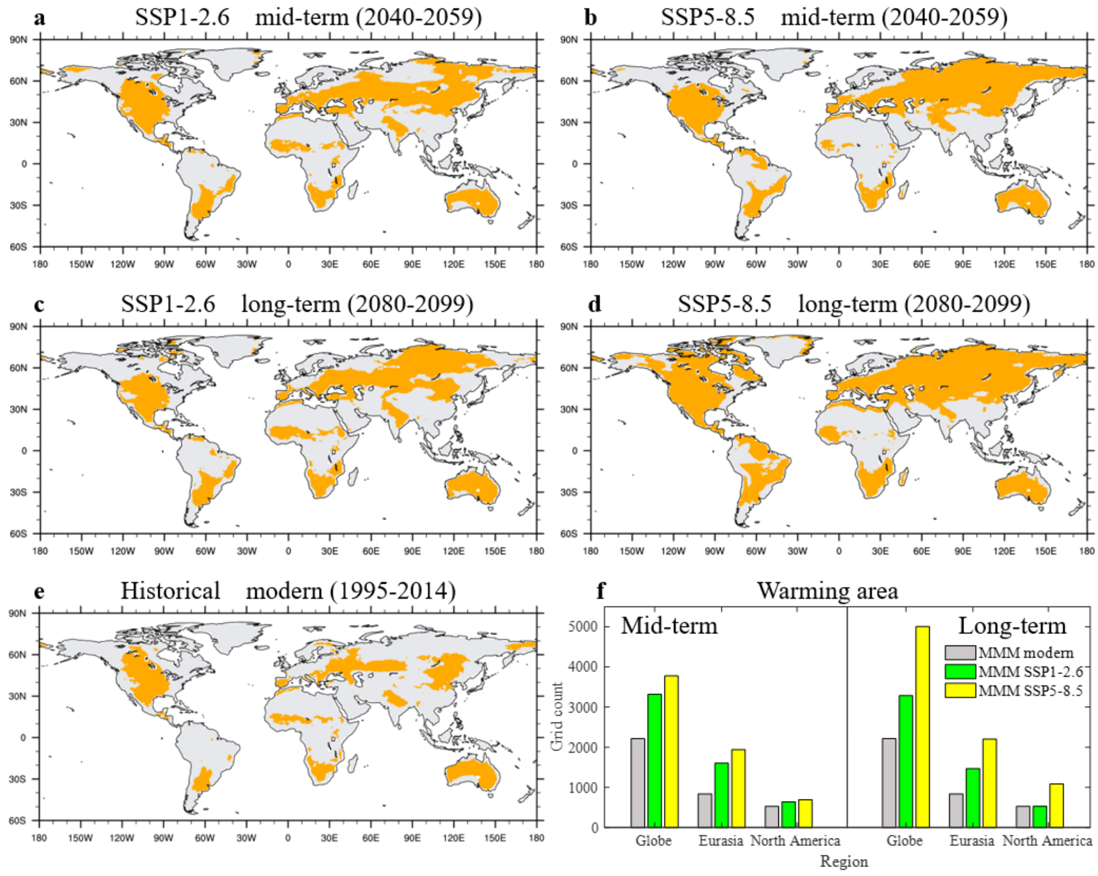

Supplementary Fig. 2| Areas in which simulated warming in the multi-model mean increased by >0.5°C owing to soil moisture-atmosphere (SA). a and **b** represent SA-driven warming exceeds 0.5°C in the mid-term future (2040–2059). **c** and **d** represent SA-driven warming exceeds 0.5°C in the long-term future (2080–2099). **a** and **c** represent output under the low-emission scenario (SSP1-2.6), and **b** and **d** represent output under the high-emission scenario (SSP5-8.5). **e** represents SA-driven warming exceeds 0.5°C in the modern period (1995–2014). **f** represents warming grid count for regions where the SA-driven rise in surface air temperature exceeds 0.5°C in the mid-term and long-term future over globe (excluding Antarctica), Eurasia, and North America.

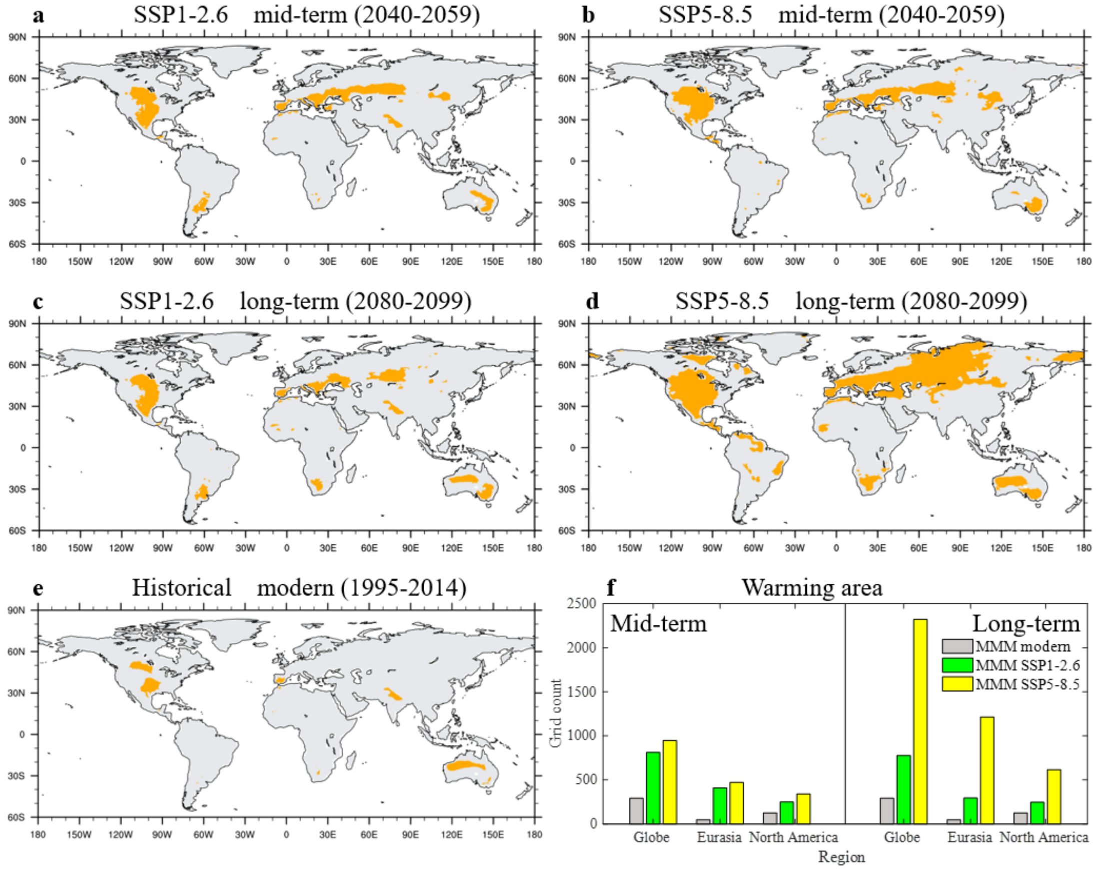

Supplementary Fig. 3| Same as Supplementary Fig. 2, but for the multi-model mean increased by >1.0°C owing to soil moisture-atmosphere.

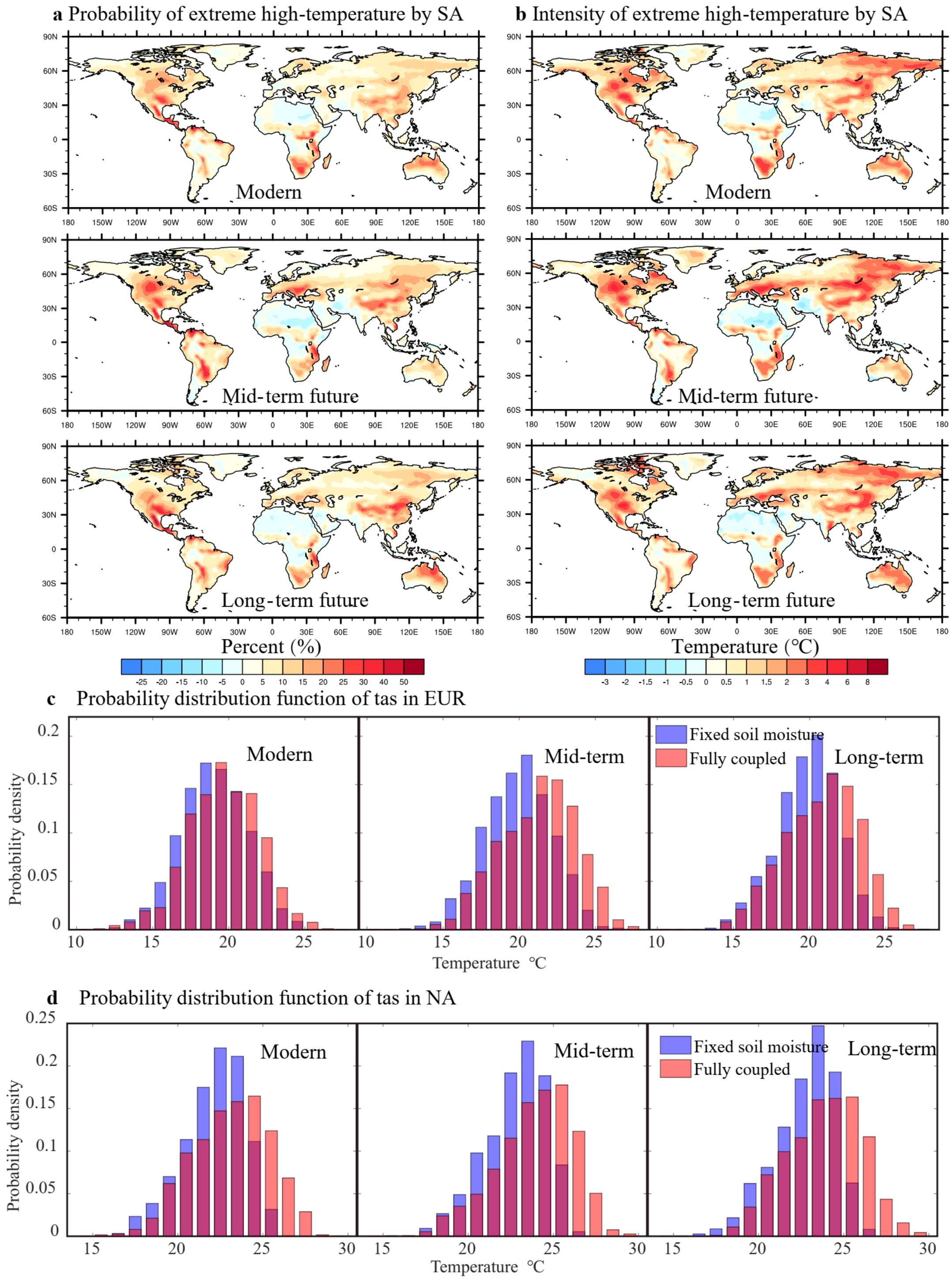

Supplementary Fig. 4| Same as Fig. 3, but for the MPI-ESM1-2-LR model under the low-emission scenario.

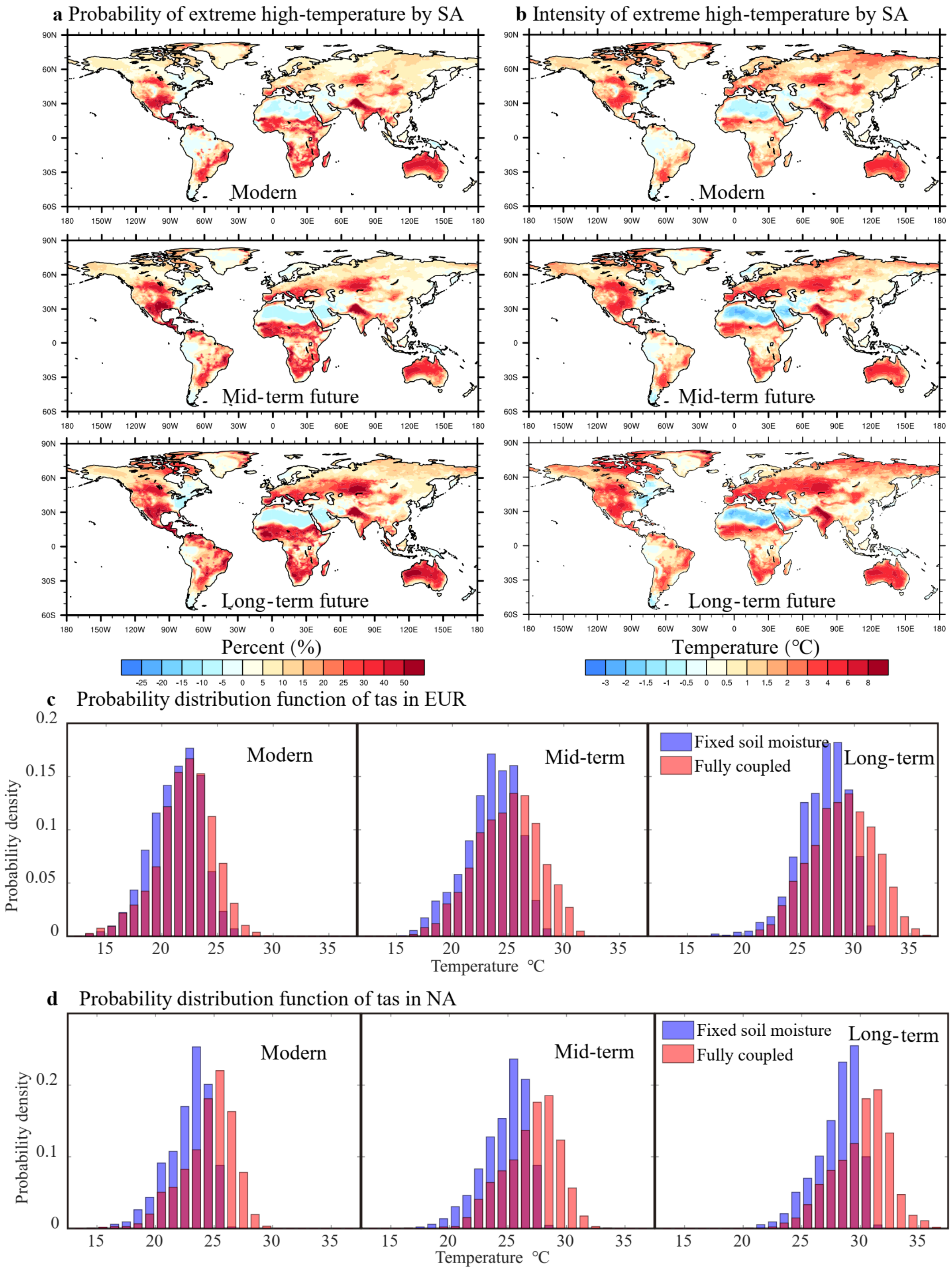

Supplementary Fig. 5| Same as Fig. 3, but for the CMCC-ESM2 model under the very high-emission scenario.

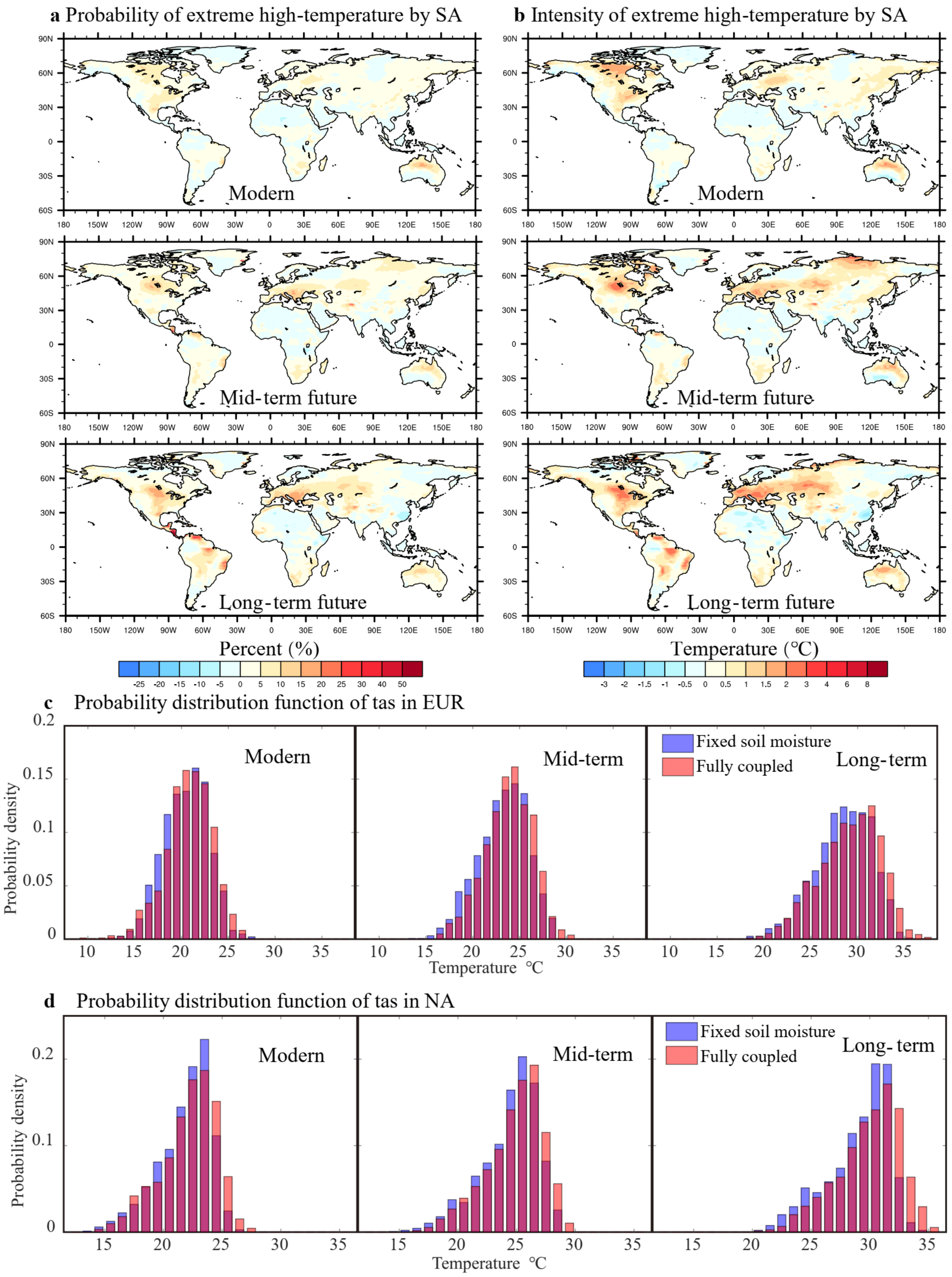

Supplementary Fig. 6| Same as Fig. 3, but for the IPSL-CM6A-LR model under the very high-emission scenario.

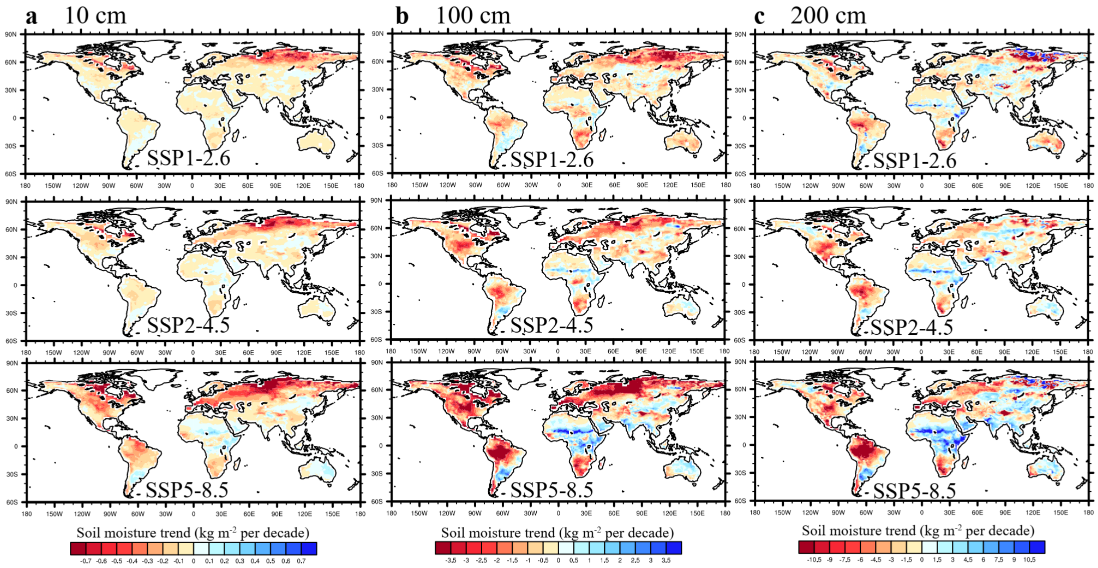

Supplementary Fig. 7| Spatial distributions of the soil moisture trend (2015-2099, kg m^{-2} per decade) under high-, mid-, and low-emission scenarios (SSP1-2.6, SSP2-4.5, and SSP5-8.5). **a**, **b**, and **c** represent soil depths are shallow (10 cm), middle (100 cm), and deep (200 cm), respectively.

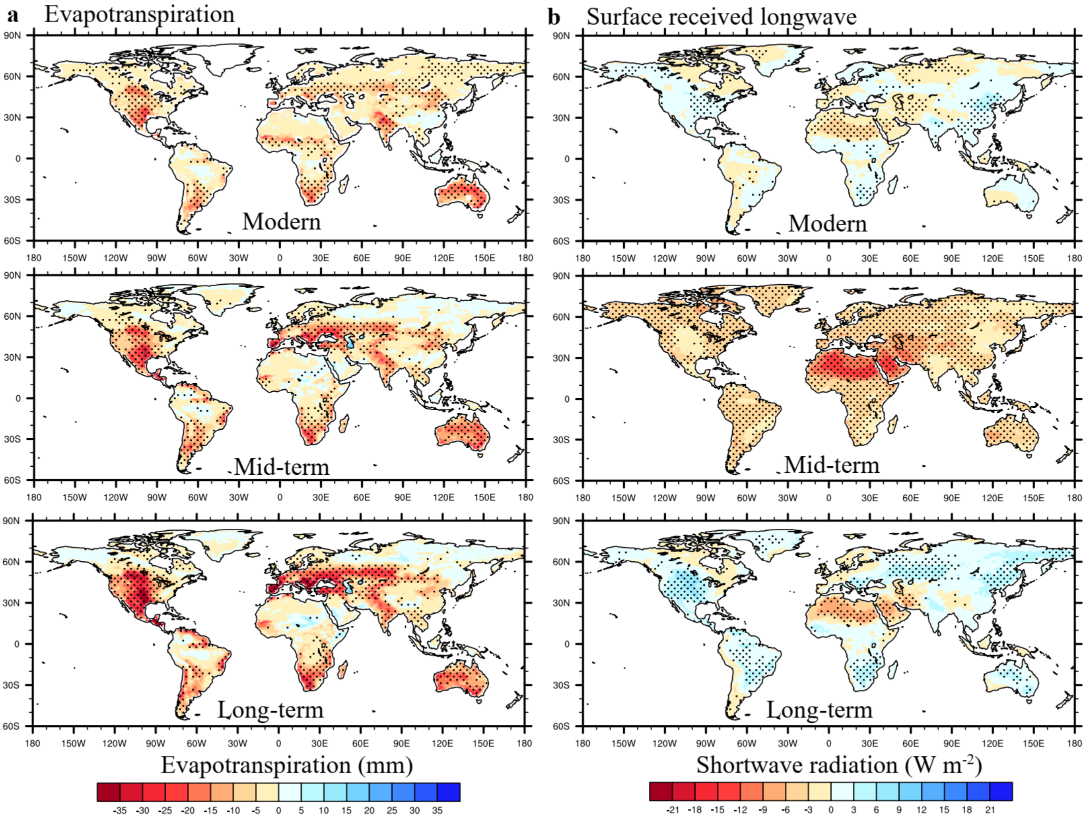

Supplementary Fig. 8| Same as Fig. 5, but for evapotranspiration (mm) and surface received longwave (W m^{-2})

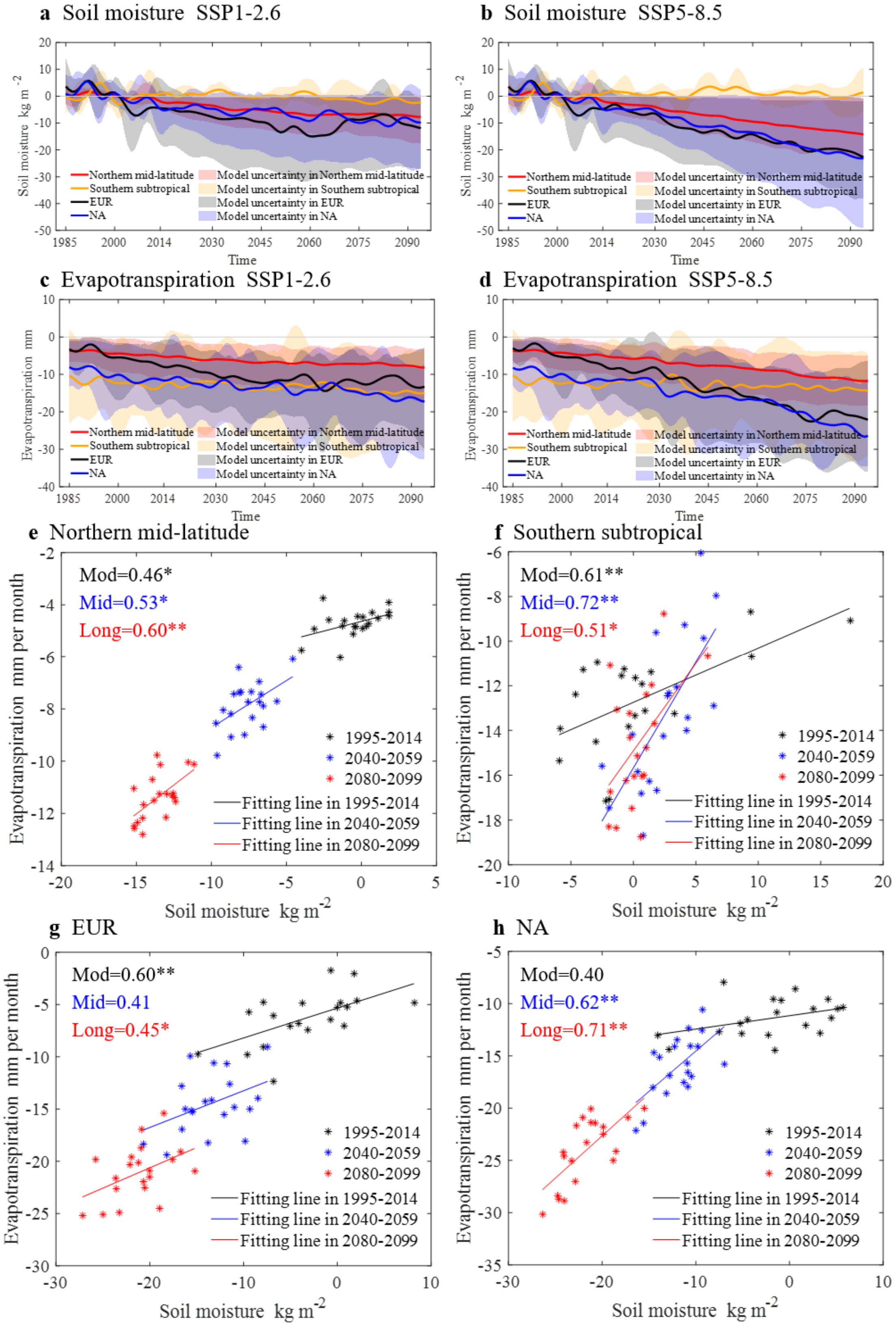

Supplementary Fig. 9| Same as Fig. 4, but for the soil moisture depth is 100 cm.

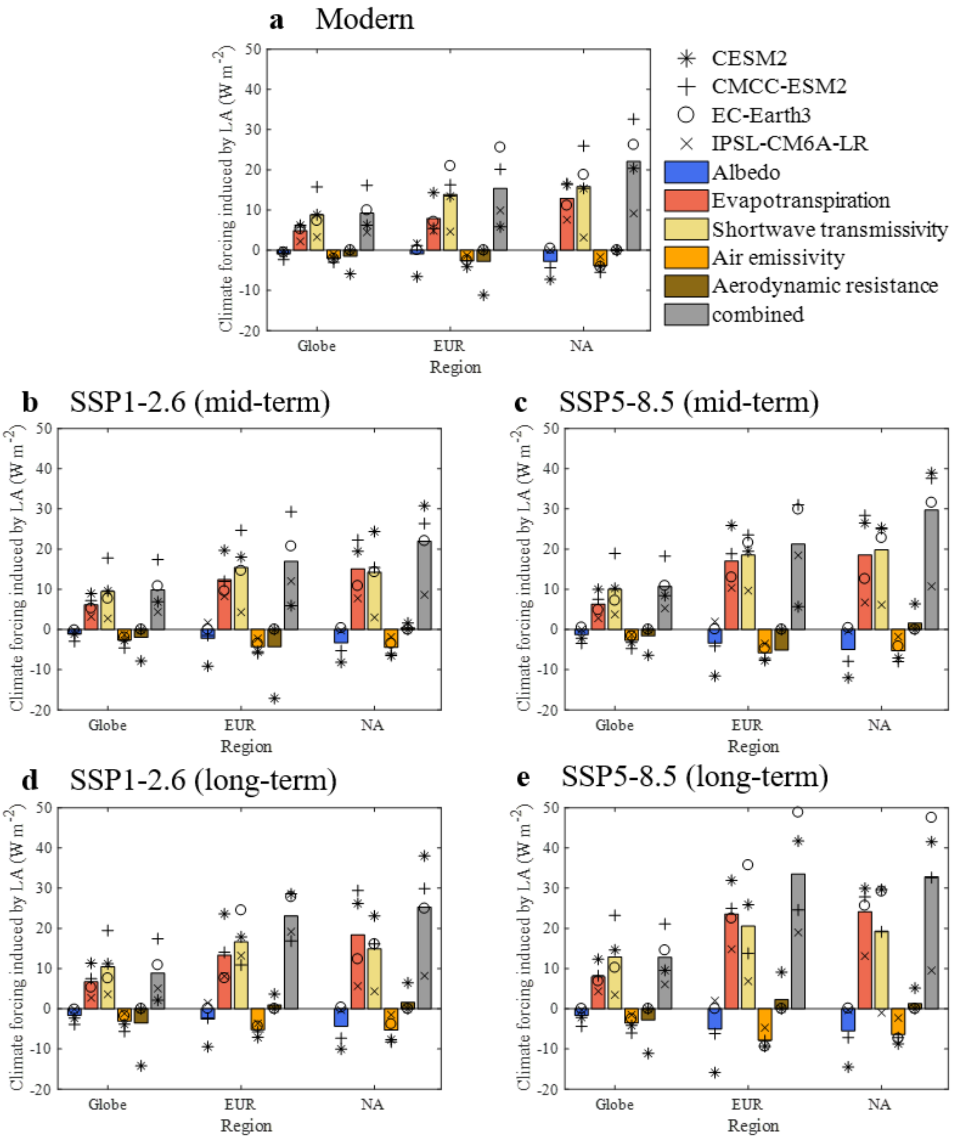

Supplementary Fig. 10| Reasons for soil moisture (SA) driven changes in surface air temperature (tas). Decomposition of tas changes caused by SA during (**a**) modern, (**b** and **c**) mid-term future, and (**d** and **e**) long-term future periods. SA-driven changes in tas are decomposed into five radiation terms (W m^{-2}) covering the globe (excluding Antarctica), EUR (40–60°N, 20–50°E), and NA (28–55°N, 88–110°W) for the modern (1995–2014), mid-term future (2040–2059), and long-term future (2080–2099) periods and under low- (**b** and **d**) and high-emission (**c** and **e**) scenarios. Blue, red, yellow, green, brown, and gray bars represent surface albedo, evapotranspiration, shortwave transmissivity, air emissivity, aerodynamic resistance, and combined radiation (the sum of the five terms), respectively.

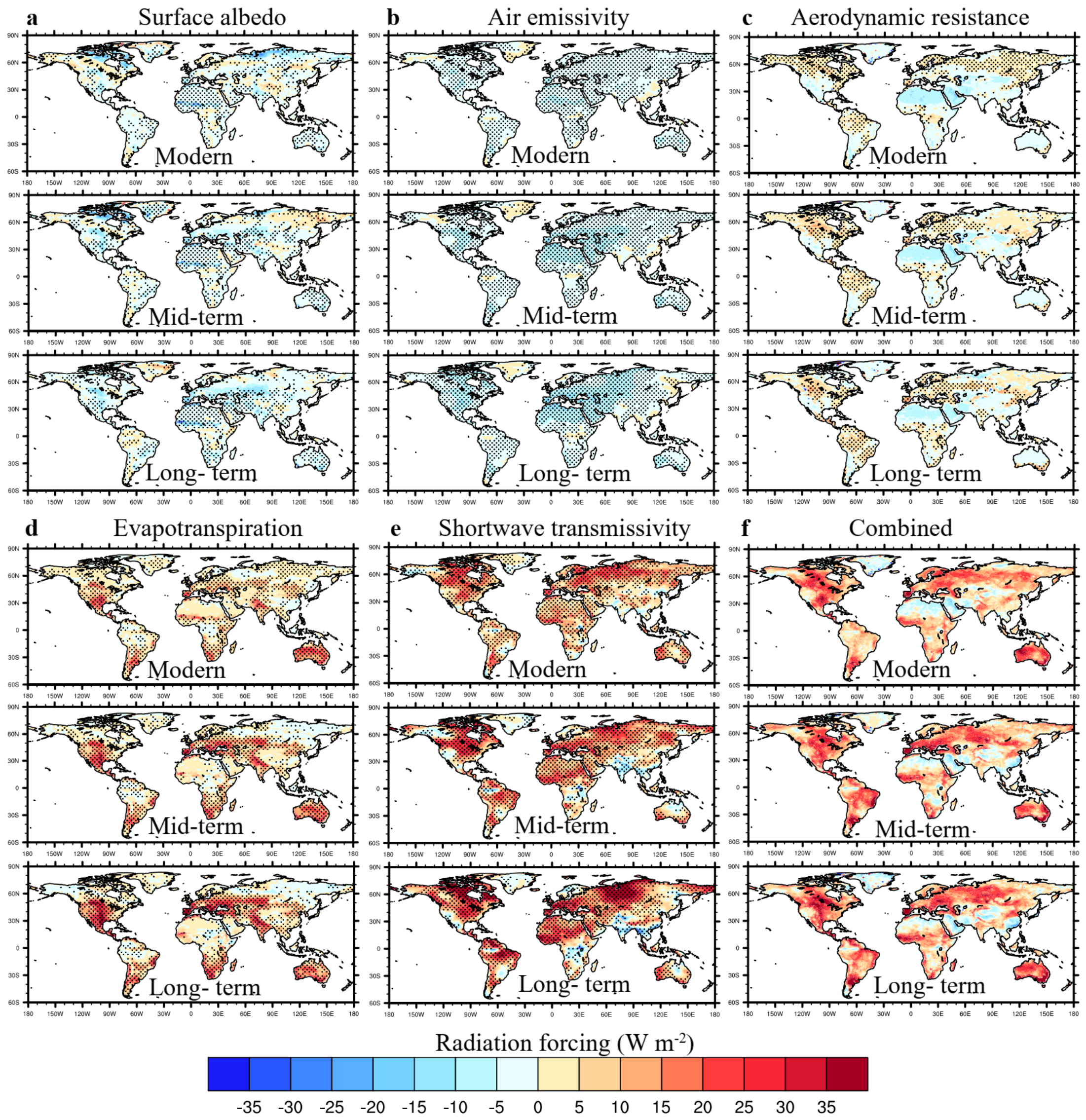

Supplementary Fig. 11| Spatial distributions of radiation forcing terms (W m^{-2}) decomposed by surface air temperature changes in modern (1995–2014), mid-term future (2040–2059), and long-term future (2080–2099) periods under the very high-emission scenario (SSP5-8.5). a is Surface albedo, **b** is air emissivity, **c** is aerodynamic resistance, **d** is evapotranspiration, **e** is shortwave transmissivity, and **f** is combined radiation (the sum of all five terms). Black dots mean that the sign of the change is consistent with that of the multi-model mean in at least three of the four CMIP6 models.
